# Supplementary material for: The destructive subterranean termite Reticulitermes flavipes (Blattodea: Rhinotermitidae) can colonize arid territories
Source: PeerJ. 2024 Feb 29;12:e16936. doi: 10.7717/peerj.16936 (PMC10909367; doi:10.7717/peerj.16936)
Supplement: Supplemental Information 1 [file peerj-12-16936-s001.docx]

**Supplemental material**

**The destructive subterranean termite *Reticulitermes flavipes* (Blattodea: Rhinotermitidae) can colonize arid territories**

**Table S1.** Meteorological stations for each zone, their coordinates and distance from the areas with *Reticulitermes flavipes.*

| **Meteorological stations** | **Zone** | **Latitude** | **Longitude** | **Distance from the focus (km)** |
| --- | --- | --- | --- | --- |
| **GC102** | Lanzarote | 29.0036111 | -13.6458333 | 5.4 |
| **LZ01** | Lanzarote | 29.0005556 | -13.5580556 | 6.4 |
| **LZ airport** | Lanzarote | 28.9519444 | -13.6002778 | 2.3 |
| **TF105** | North Tenerife | 28.5128145 | -16.3878427 | 0.7 |
| **TF106** | North Tenerife | 28.5280556 | -16.3869444 | 0 |
| **TF108** | North Tenerife | 28.5247222 | -16.3700000 | 1.5 |
| **TF109** | North Tenerife | 28.5063125 | -16.3892082 | 0.1 |
| **TF02** | South Tenerife | 28.2300000 | -16.8341667 | 0.7 |
| **TF13** | South Tenerife | 28.0406000 | -16.6532300 | 8.8 |

**Table S2.** The area occupied by *R. flavipes* each year in the northeast of Tenerife.

| **Locality** | **Main focus** | **Distal focus 1** | **Distal focus 2** | **Distal focus 3** | **Distal focus 4** |
| --- | --- | --- | --- | --- | --- |
| **Area (m^2^)/year** | Tacoronte-La Laguna | La Barranquera | Los Collazos | Garimba | Los Naranjeros |
| **2010** | 89,900 | - | - | - | - |
| **2015** | 107,116 | - | - | - | - |
| **2017** | 120,909 | - | - | - | - |
| **2018** | 949,614 | - | - | - | - |
| **2019** | 1,061,021 | - | - | - | - |
| **2020** | 2,599,891 | 19,322 | 3,005 | 11,693 | 5,285 |
| **2021** | 3,416,966 | 63,136 | - | 26,834 | 6,628 |
| **2022** | **3,669,703** | **63,474** | **-** | **29,643** | - |

**Table S3.** The area occupied by *R. flavipes* each year in Lanzarote and the south of Tenerife.

| **Locality** | **Lanzarote** | **Southern focus 1** | **Southern focus 2** |
| --- | --- | --- | --- |
| **Area (m^2^)/year** | Güíme | Los Cristianos | Puerto Santiago |
| **2018** | - | 15 | - |
| **2019** | - | 15 | - |
| **2020** | 10,942 | 8,233 | 13,748 |
| **2021** | 11,678 | 9,143 | 18,306 |
| **2022** | **21,377** | **37,043** | **19,972** |

**Table S4.** Expansion of *R. flavipes* in each of its populations in the north-east of Tenerife. Maximum, minimum, and mean linear distance of advance by *R. flavipes* between 2010-2022. The main direction of their advance is also indicated.

| **Linear expansion (m)/year** | | **Main focus** | **Distal focus 1** | **Distal focus 3** | **Distal focus 4** |
| --- | --- | --- | --- | --- | --- |
|  |  | **Tacoronte-La Laguna** | **La Barranquera** | **Garimba** | **Los Naranjeros** |
| **2010-2015** | **Min.** | 5.44 | - | - | - |
|  | **Max.** | 183.25 | - | - | - |
|  | **Mean** | **79.5** | - | - | - |
|  | **Main direction** | South | - | - | - |
| **2015-2017** | **Min.** | 6.29 | - | - | - |
|  | **Max.** | 133.66 | - | - | - |
|  | **Mean** | **63.87** | - | - | - |
|  | **Main direction** | West | - | - | - |
| **2017-2018** | **Min.** | 5.09 | - | - | - |
|  | **Max.** | 866.5 | - | - | - |
|  | **Mean** | **407.13** | - | - | - |
|  | **Main direction** | East | - | - | - |
| **2018-2019** | **Min.** | 5.04 | - | - | - |
|  | **Max.** | 140.21 | - | - | - |
|  | **Mean** | **53.34** | - | - | - |
|  | **Main direction** | Northwest | - | - | - |
| **2019-2020** | **Min.** | 5.53 | - | - | - |
|  | **Max.** | 1046.97 | - | - | - |
|  | **Mean** | **434.08** | - | - | - |
|  | **Main direction** | East | - | - | - |
| **2020-2021** | **Min.** | 5.06 | 5 | 5.94 | 7.05 |
|  | **Max.** | 1027.13 | 332.83 | 122.4 | 25.61 |
|  | **Mean** | **258.49** | **152.82** | **53.65** | **18.61** |
|  | **Main direction** | North | Northwest | North | West |
| **2021-2022** | **Min.** | 5.02 | - | 5.08 | - |
|  | **Max.** | 169.31 | - | 38.3 | - |
|  | **Mean** | **39.25** | - | **23.76** | - |
|  | **Main direction** | Southeast | - | Northeast | - |

**Table S5.** Expansion of *R. flavipes* in each population in Lanzarote and the south of Tenerife. Maximum, minimum, and mean linear distance of advance by *R. flavipes* between 2020-2022. In addition, the main direction of advance is indicated.

| **Linear expansion (m)/year** | | **Lanzarote** | **Southern focus 1** | **Southern focus 2** |
| --- | --- | --- | --- | --- |
|  |  | **Güíme** | **Los Cristianos** | **Puerto Santiago** |
| **2020-2021** | **Min.** | 6.37 | 6.00 | 5.43 |
|  | **Max.** | 16.08 | 15.44 | 46.96 |
|  | **Mean** | **10.64** | **12.09** | **20.19** |
|  | **main direction** | South | South | East |
| **2021-2022** | **Min.** | 6.04 | 5.11 | 5.37 |
|  | **Max.** | 59.03 | 414.87 | 24.11 |
|  | **Mean** | **31.63** | **213.91** | **12.62** |
|  | **main direction** | East | North | Northeast |

**Table S6.** Pairwise comparisons of climatic variables between the three zones. NT: North Tenerife; ST: South Tenerife; LZ: Lanzarote.

| **Pairwise comparisons** | | | |
| --- | --- | --- | --- |
| **Mean Temperature** | | | |
|  | **Estimate** | **SE** | ***p*** |
| NT-LZ | -2.25381 | 0.26191 | < 1x10^-5^ |
| NT-ST | 2.32967 | 0.29555 | < 1x10^-5^ |
| ST-LZ | 0.07586 | 0.31259 | 0.968 |
| **Max. Temperature** | | | |
| NT-LZ | -2.0939 | 0.4181 | < 1x10^-5^ |
| NT-ST | 2.4381 | 0.4719 | < 1x10^-5^ |
| ST-LZ | 0.3442 | 0.4991 | 0.769 |
| **Min. Temperature** | | | |
| NT-LZ | -2.3236 | 0.2998 | < 1x10^-4^ |
| NT-ST | 1.7661 | 0.3383 | < 1x10^-4^ |
| ST-LZ | -0.5575 | 0.3578 | 0.263 |
| **Mean Humidity** | | | |
| NT-LZ | -0.06164 | 0.73277 | 0.996 |
| NT-ST | -4.37508 | 0.82554 | < 1x10^-6^ |
| ST-LZ | -4.43673 | 0.82554 | < 1x10^-6^ |
| **Precipitation** | | | |
| NT-LZ | 22.031 | 2.681 | < 1x10^-4^ |
| NT-ST | -25.793 | 3.026 | < 1x10^-4^ |
| ST-LZ | -3.762 | 3.200 | 0.466 |

**Table S7.** Differences in climatic variables between the three zones in each season. NT: North Tenerife; ST: South Tenerife; LZ: Lanzarote.

| **Zones/Seasons** | | | | | | |
| --- | --- | --- | --- | --- | --- | --- |
| **SPRING** | | | | | | |
|  | **χ^2^** | ***df*** | ***p*** | **Mean ± SE NT** | **Mean ± SE ST** | **Mean ± SE LZ** |
| **Mean Temperature** | 94.621 | 2 | 2.2x10^-16^ | 16.5 ± 0.195 | 19.0 ± 0.276 | 19.1 ± 0.228 |
| **Max. Temperature** | 16.393 | 2 | 2.7x10^-4^ | 24.6 ± 0.489 | 27.2 ± 0.691 | 27.4 ± 0.571 |
| **Min. Temperature** | 44.662 | 2 | 2.00x10^-10^ | 11.3 ± 0.254 | 13.2 ± 0.359 | 13.7 ± 0.297 |
| **Mean Humidity** | 22.453 | 2 | 1.33x10^-5^ | 71.6 ± 0.650 | 66.3 ± 0.931 | 68.9 ± 0.769 |
| **Precipitation** | 102.3 | 2 | 2.2x10^-16^ | 33.56 ± 2.09 | 4.71 ± 2.96 | 5.32 ± 2.44 |
| **SUMMER** | | | | | | |
| **Mean Temperature** | 95.321 | 2 | 2.2x10^-16^ | 20.4 ± 0.198 | 22.9 ±0.280 | 23.0 ± 0.229 |
| **Max. Temperature** | 20.724 | 2 | 3.16x10^-5^ | 28.7 ± 0.485 | 31.1 ± 0.686 | 32.0 ± 0.560 |
| **Min. Temperature** | 74.725 | 2 | 2.2x10^-16^ | 15.4 ± 0.236 | 17.3 ± 0.334 | 18.4 ± 0.272 |
| **Mean Humidity** | 7.4007 | 2 | 0.02471 | 74.2 ± 0.760 | 70.8 ± 1.075 | 72.2 ± 0.878 |
| **Precipitation** | 37.981 | 2 | 5.65x10^-9^ | 11.93 ± 1.27 | 1.96 ± 1.79 | 1.15 ± 1.46 |
| **AUTUMN** | | | | | | |
| **Mean Temperature** | 54.128 | 2 | 1.76x10^-12^ | 20.1 ± 0.220 | 22.3 ± 0.311 | 22.2 ± 0.254 |
| **Max. Temperature** | 10.351 | 2 | 0.005654 | 28.5 ± 0.439 | 30.5 ± 0.621 | 30.3 ± 0.507 |
| **Min. Temperature** | 25.442 | 2 | 2.98x10^-6^ | 14.8 ± 0.284 | 16.5 ± 0.402 | 16.8 ± 0.328 |
| **Mean Humidity** | 3.7961 | 2 | 0.1499 | 70.1 ± 0.983 | 67.4 ± 1.390 | 70.8 ± 1.148 |
| **Precipitation** | 15.861 | 2 | 3.5x10^-4^ | 42.9 ± 5.42 | 10.2 ± 7.66 | 17.2 ± 6.26 |
| **WINTER** | | | | | | |
| **Mean Temperature** | 48.341 | 2 | 3.18x10^-11^ | 15.9 ± 0.193 | 18.1 ± 0.272 | 17.3 ± 0.228 |
| **Max. Temperature** | 22.878 | 2 | 1.07x10^-5^ | 23.6 ± 0.339 | 26.3 ± 0.480 | 24.0 ± 0.401 |
| **Min. Temperature** | 20.92 | 2 | 2.86x10^-5^ | 10.5 ± 0.246 | 11.9 ± 0.348 | 12.0 ± 0.291 |
| **Mean Humidity** | 32.787 | 2 | 7.59x10^-8^ | 64.2 ± 0.963 | 58.1 ± 1.362 | 38.3 ± 1.138 |
| **Precipitation** | 48.964 | 2 | 2.33x10^-11^ | 40.32 ± 2.90 | 8.66 ± 4.10 | 17.12 ± 3.43 |

**Table S8.** Pairwise comparisons of climatic variables between the three zones in each season. NT: North Tenerife; ST: South Tenerife; LZ: Lanzarote.

| **Pairwise comparisons Zones/Season** | | | |
| --- | --- | --- | --- |
| **SPRING** | | | |
| **Mean Temperature** | | | |
|  | **Estimate** | **SE** | ***p*** |
| NT-LZ | -2.58591 | 0.29980 | < 1x10^-5^ |
| NT-ST | 2.50167 | 0.33775 | < 1x10^-5^ |
| ST-LZ | -0.08425 | 0.35764 | 0.97 |
| **Max. Temperature** | | | |
| NT-LZ | -2.7195 | 0.7514 | 0.00106 |
| NT-ST | 2.5745 | 0.8963 | 0.00659 |
| ST-LZ | -0.1450 | 0.8465 | 0.98562 |
| **Min. Temperature** | | | |
| NT-LZ | -2.4649 | 0.3904 | < 1x10^-4^ |
| NT-ST | 1.9353 | 0.4398 | < 1x10^-4^ |
| ST-LZ | -0.5296 | 0.4657 | 0.49 |
| **Mean Humidity** | | | |
| NT-LZ | 2.652 | 1.012 | 0.0235 |
| NT-ST | -5.293 | 1.140 | <0.001 |
| ST-LZ | -2.641 | 1.207 | 0.0726 |
| **Precipitation** | | | |
| NT-LZ | 28.2354 | 3.2180 | < 1x10^-6^ |
| NT-ST | -28.8508 | 3.6254 | < 1x10^-6^ |
| ST-LZ | -0.6155 | 3.8389 | 0.986 |
| **SUMMER** | | | |
| **Mean Temperature** | | | |
| NT-LZ | -2.6635 | 0.3025 | < 1x10^-5^ |
| NT-ST | 2.4818 | 0.3430 | < 1x10^-5^ |
| ST-LZ | -0.1817 | 0.3616 | 0.87 |
| **Max. Temperature** | | | |
| NT-LZ | -3.2316 | 0.7409 | <0.001 |
| NT-ST | 2.3990 | 0.8401 | 0.012 |
| ST-LZ | -0.8326 | 0.8855 | 0.613 |
| **Min. Temperature** | | | |
| NT-LZ | -3.0558 | 0.3603 | <0.001 |
| NT-ST | 1.9423 | 0.4086 | <0.001 |
| ST-LZ | -1.1134 | 0.4307 | 0.0263 |
| **Mean Humidity** | | | |
| NT-LZ | 1.976 | 1.161 | 0.2035 |
| NT-ST | -3.435 | 1.317 | 0.0244 |
| ST-LZ | -1.459 | 1.388 | 0.5431 |
| **Precipitation** | | | |
| NT-LZ | 10.786 | 1.936 | < 1x10^-5^ |
| NT-ST | -9.974 | 2.195 | 1.46x10^-5^ |
| ST-LZ | 0.812 | 2.314 | 0.934 |
| **AUTUMN** | | | |
| **Mean Temperature** | | | |
| NT-LZ | -2.172949 | 0.336419 | < 1x10^-7^ |
| NT-ST | 2.175333 | 0.381463 | < 1x10^-7^ |
| ST-LZ | 0.002384 | 0.402097 | 1 |
| **Max. Temperature** | | | |
| NT-LZ | -1.7868 | 0.6705 | 0.0209 |
| NT-ST | 2.0343 | 0.7602 | 0.0202 |
| ST-LZ | 0.2476 | 0.8013 | 0.9485 |
| **Min. Temperature** | | | |
| NT-LZ | -2.0207 | 0.4341 | <0.001 |
| NT-ST | 1.7517 | 0.4922 | 0.00108 |
| ST-LZ | -0.2690 | 0.5189 | 0.86174 |
| **Mean Humidity** | | | |
| NT-LZ | -0.7121 | 1.5116 | 0.884 |
| NT-ST | -2.6825 | 1.7029 | 0.143 |
| ST-LZ | -3.3946 | 1.8032 | 0.255 |
| **Precipitation** | | | |
| NT-LZ | 25.702 | 8.278 | 0.00523 |
| NT-ST | -32.689 | 9.386 | 0.00134 |
| ST-LZ | -6.987 | 9.894 | 0.75886 |
| **WINTER** | | | |
| **Mean Temperature** | | | |
| NT-LZ | -1.4225 | 0.2982 | <0.001 |
| NT-ST | 2.1598 | 0.3337 | <0.001 |
| ST-LZ | 0.7374 | 0.3550 | 0.094 |
| **Max. Temperature** | | | |
| NT-LZ | -0.4163 | 0.5249 | 0.706231 |
| NT-ST | 2.7447 | 0.5874 | < 1x10^-4^ |
| ST-LZ | 2.3284 | 0.6250 | 0.000571 |
| **Min. Temperature** | | | |
| NT-LZ | -1.5730 | 0.3810 | <0.001 |
| NT-ST | 1.4352 | 0.4264 | 0.00223 |
| ST-LZ | -0.1379 | 0.4536 | 0.95012 |
| **Mean Humidity** | | | |
| NT-LZ | -4.072 | 1.491 | 0.0172 |
| NT-ST | -6.091 | 1.669 | <0.001 |
| ST-LZ | -10.163 | 1.775 | <0.001 |
| **Precipitation** | | | |
| NT-LZ | 23.198 | 4.488 | < 1x10^-4^ |
| NT-ST | -31.660 | 5.022 | < 1x10^-4^ |
| NT-LZ | -8.461 | 5.343 | 0.251 |

**Figures**

**
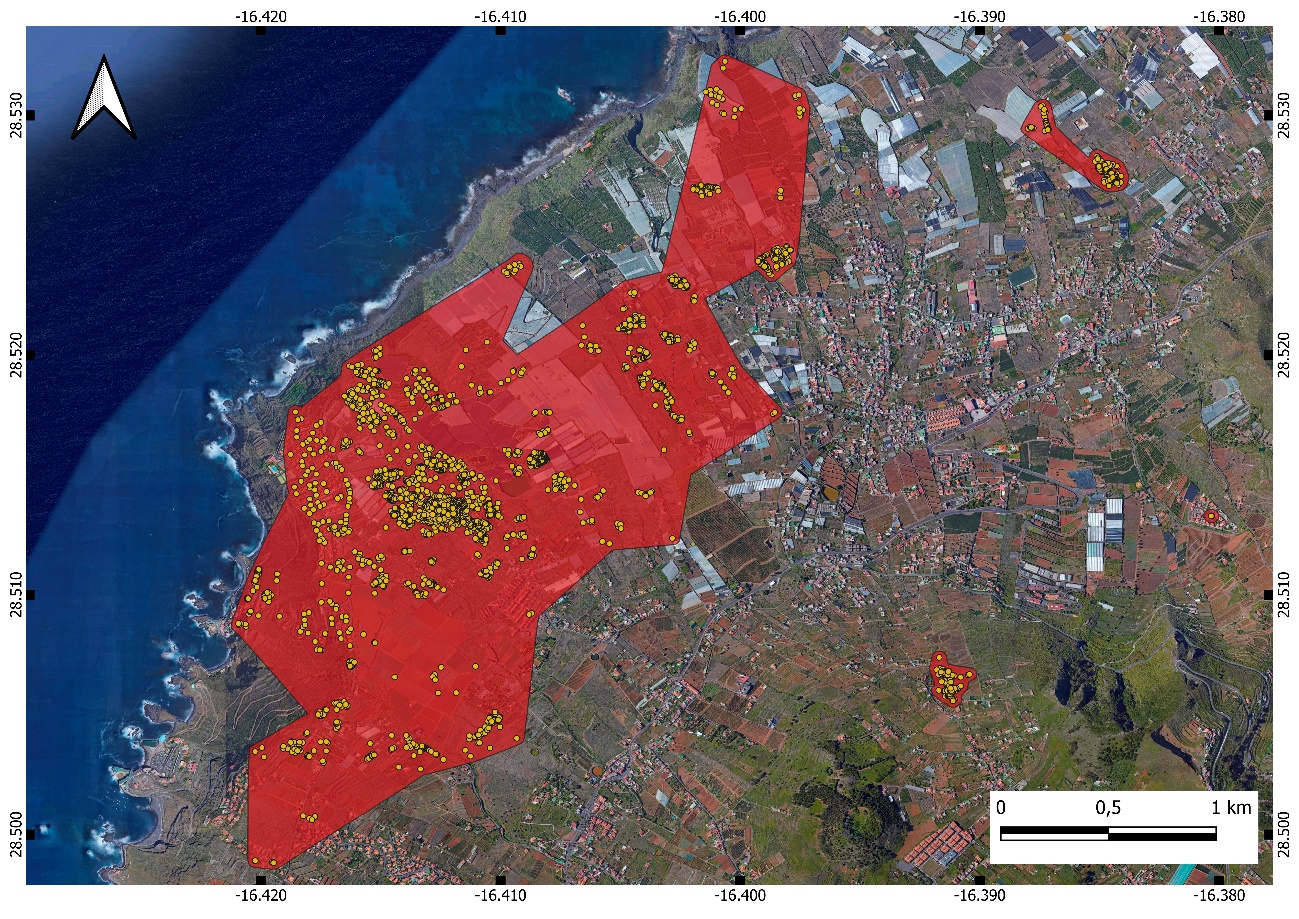
**

**Figure S1.** Detailed map with *R. flavipes* distribution (yellow dots and red area) in the main focus on Tenerife. Map credit: Gobierno de Canarias.

**
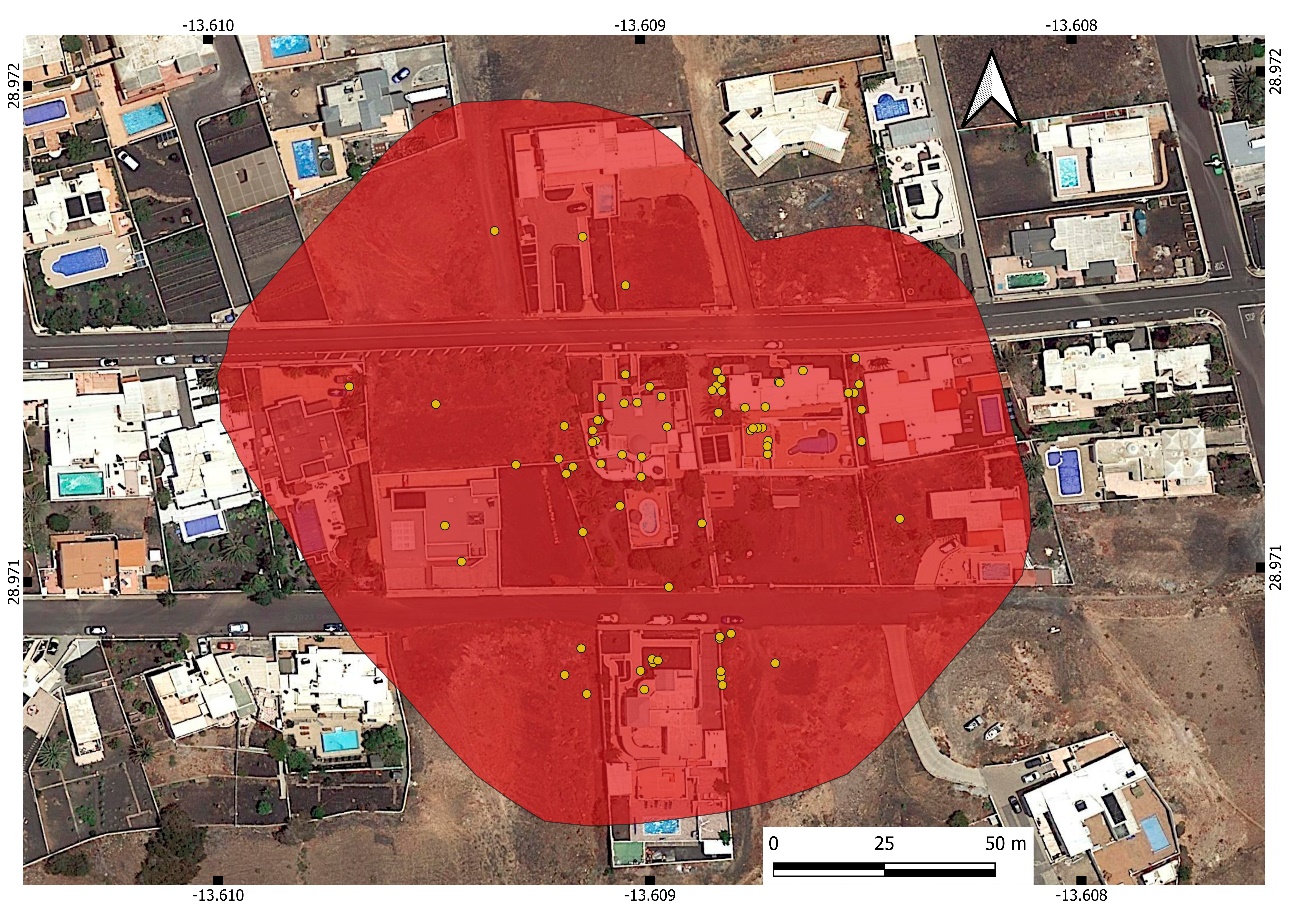
**

**Figure S2.** Detailed map with *R. flavipes* distribution (yellow dots and red area) in Lanzarote. Map credit: Gobierno de Canarias.

**
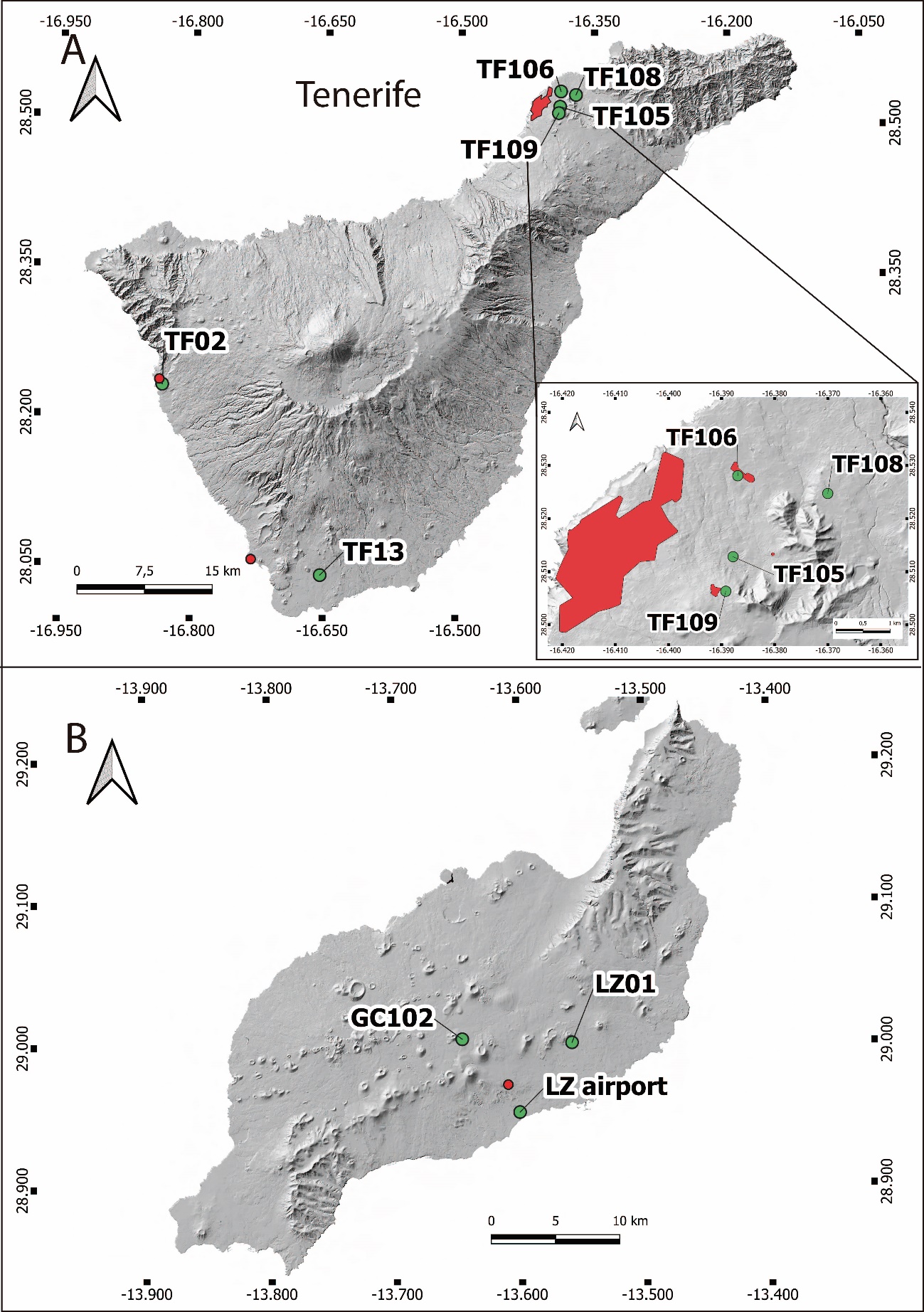
**

**Figure S3.** Map with *R. flavipes* distribution (red dots and area) on Tenerife (A) and Lanzarote (B), and the location of the meteorological stations used (green points). Map credit: Gobierno de Canarias.

**
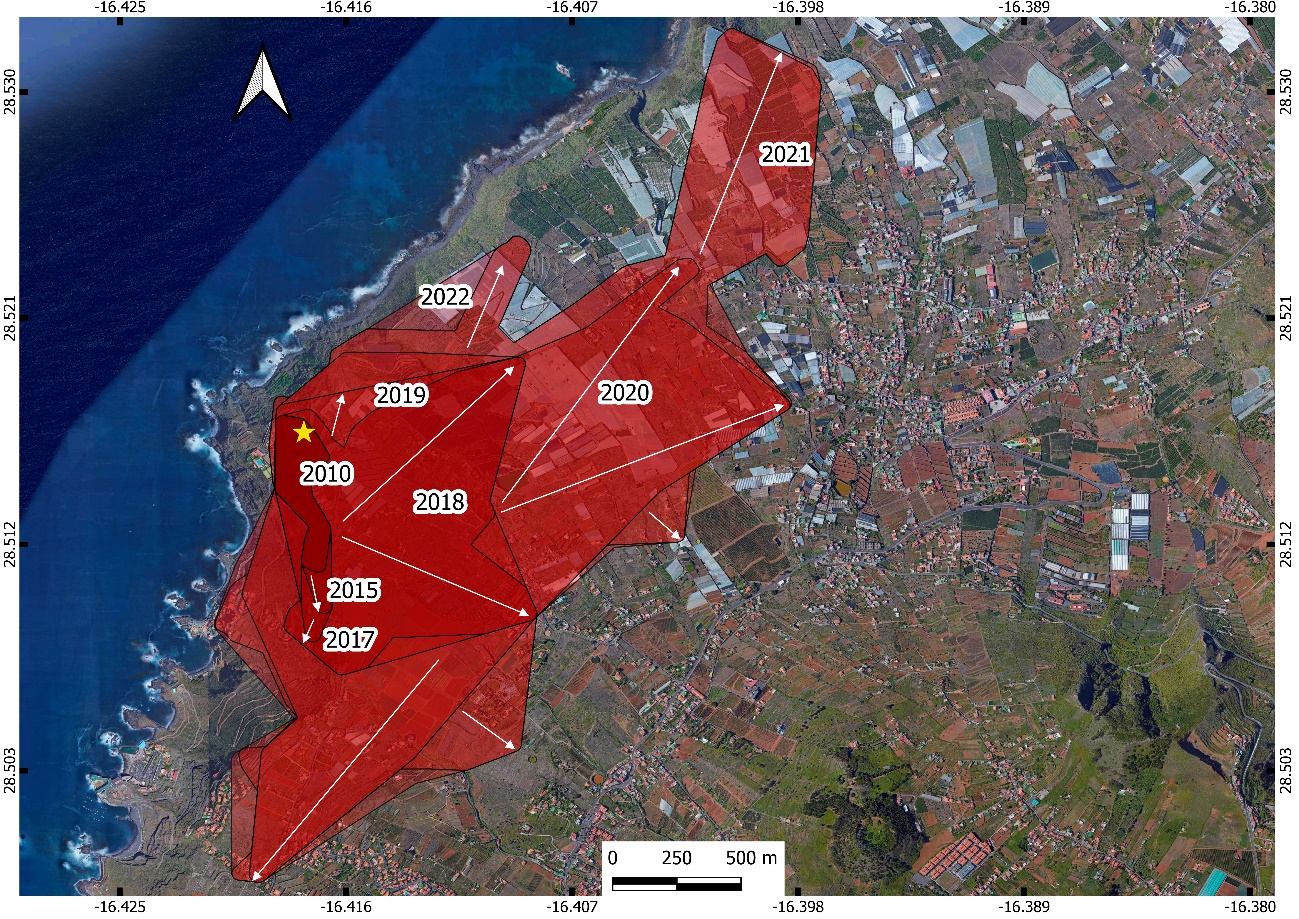
**

**Figure S4.** Map with *R. flavipes* annual distribution in the main focus of Tenerife from 2009 to 2022. The areas are represented by a scale of red, from deepest to lightest. White arrows represent the main fronts of progress in each year. A star indicates the first point where *R. flavipes* was detected. Map credit: Gobierno de Canarias.


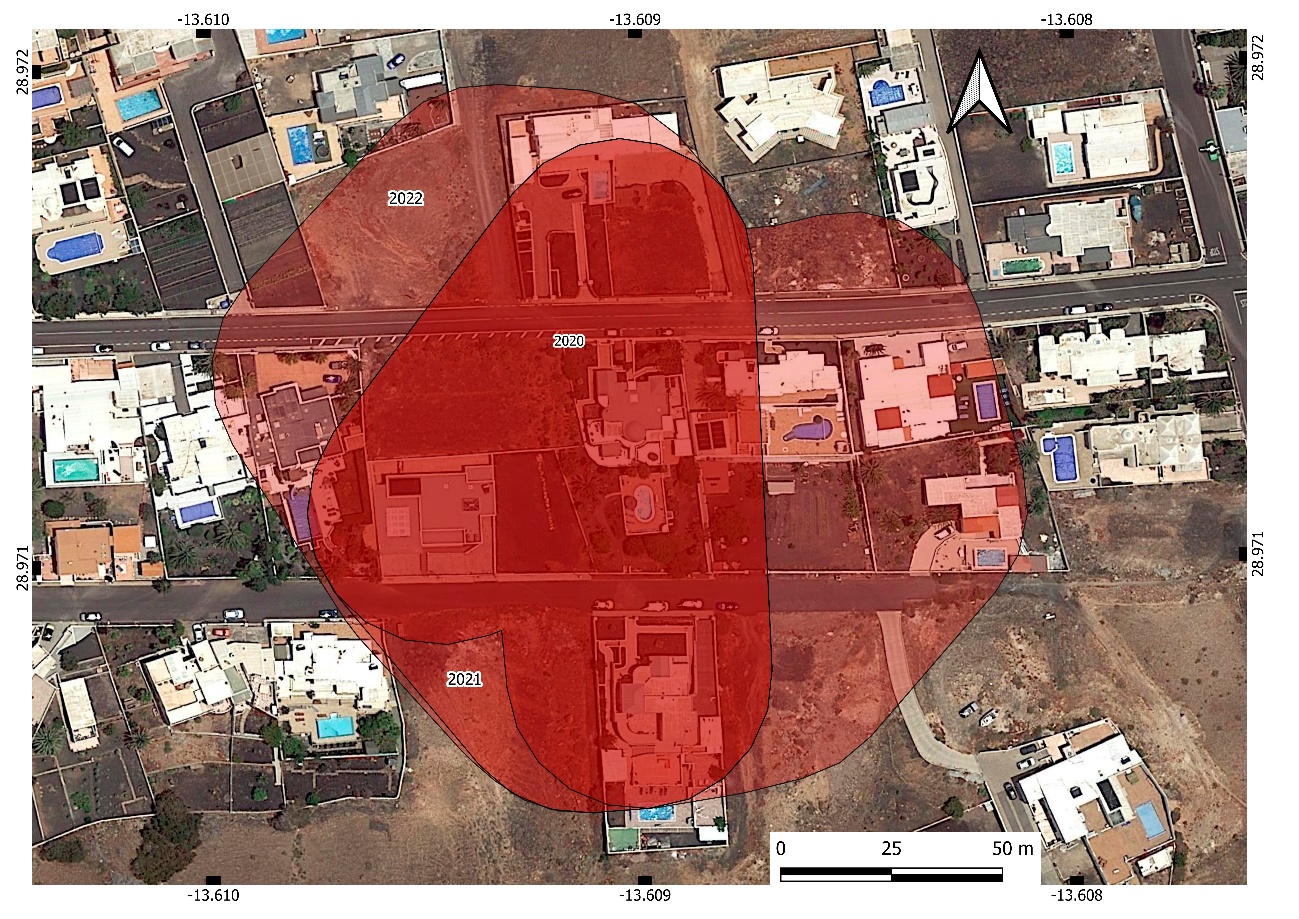


**Figure S5.** Map with *R. flavipes* annual distribution in the Lanzarote focus from 2020 to 2022. The areas are represented by a scale of red, from deepest to lightest. Map credit: Gobierno de Canarias.

**..**


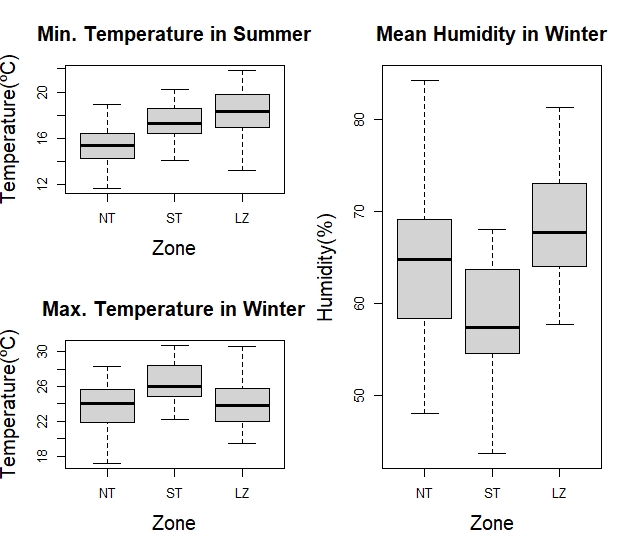


**Figure S6.** Differences between South Tenerife and Lanzarote in Minimum Temperature in summer and Maximum Temperature and Mean Humidity in winter. The boxes comprise 50% of the data, the black horizontal lines represent the median, and the whiskers extend to the last value within 1.5 times the interquartile range. Abbreviations: NT, North Tenerife; ST, South Tenerife; and LZ, Lanzarote.

,**Figure S7.** World map representing the zones with less annual rainfall than Lanzarote and with a suitable mean temperature for the establishment of *Reticulitermes flavipes* (grey) and the worldwide distribution of this species (orange dots). Map data © EuroGeographics for the administrative boundaries.


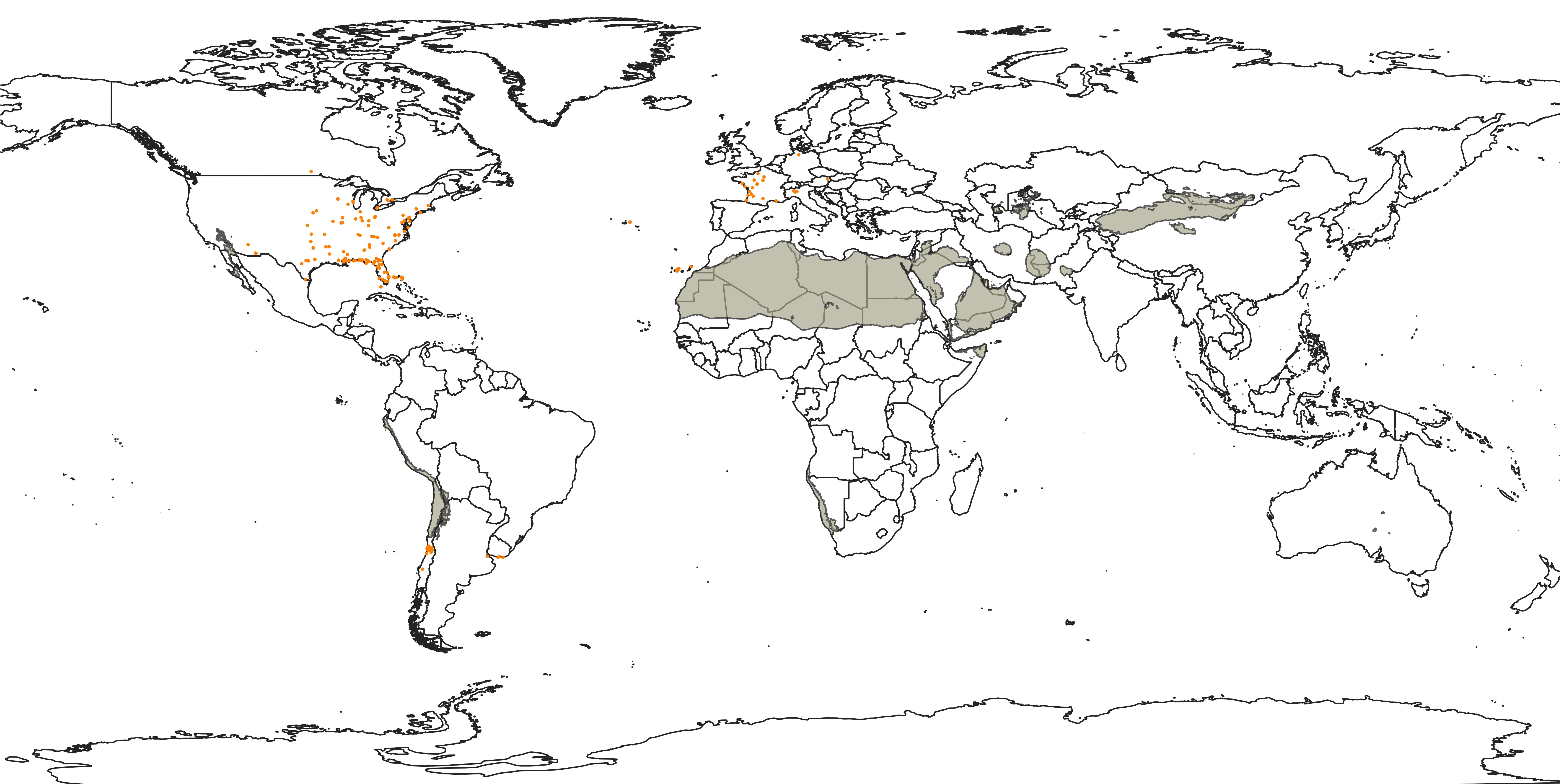

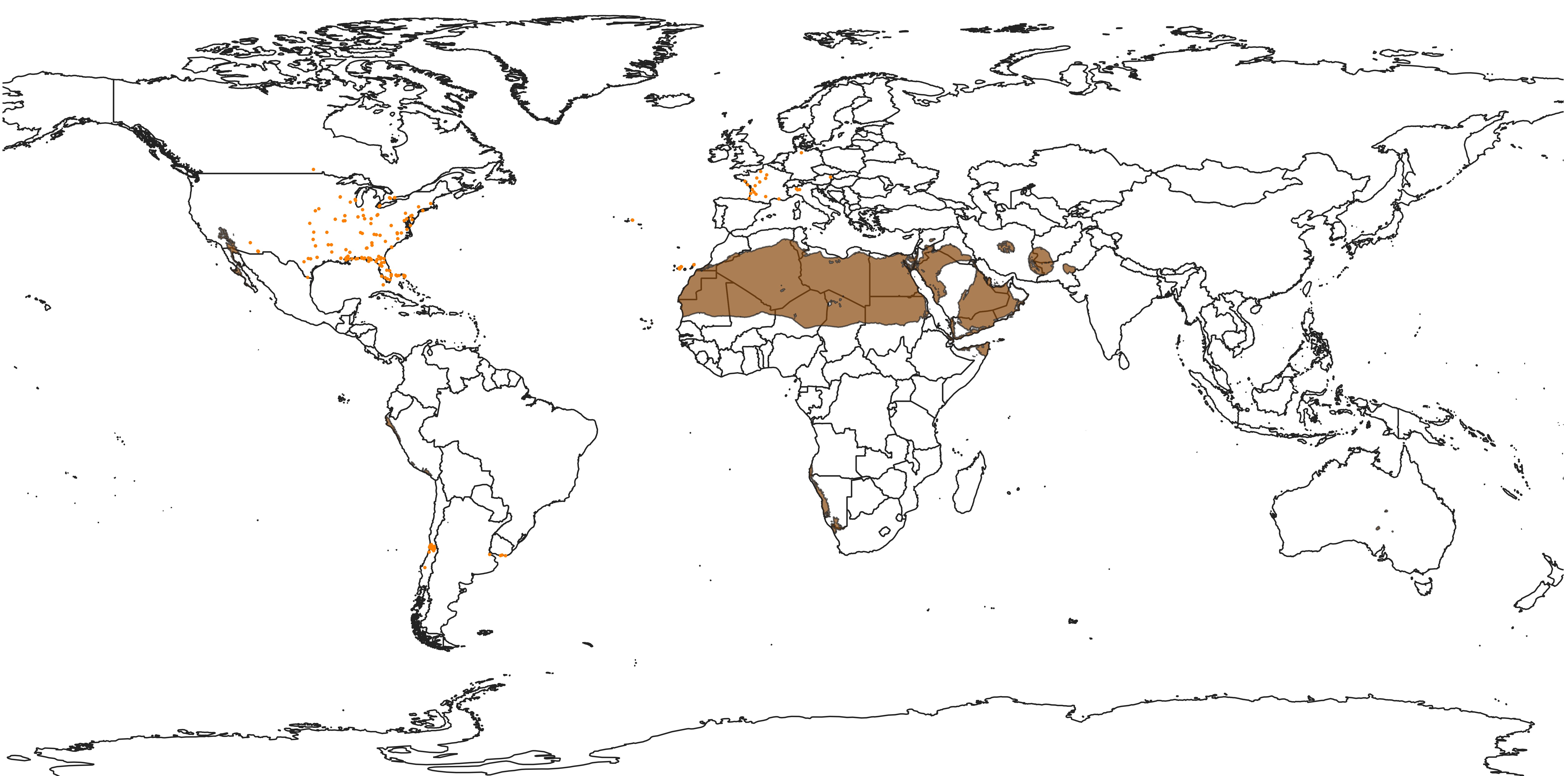


**Figure S8.** World map representing the zones with less annual rainfall and higher mean temperature than Lanzarote (brown) and the worldwide distribution of this species (orange dots). Map data © EuroGeographics for the administrative boundaries.
